# Outcomes of a food voucher program and factors associated with the recovery rate of children with moderate acute malnutrition in Far North Cameroon

**DOI:** 10.1186/s41043-023-00379-1

**Published:** 2023-04-29

**Authors:** Ismael Teta, Brice Ulrich Saha Foudjo, Jennifer N. Nielsen, Julius Oben, Georges Nguefack-Tsague, Françoise Raissa Ntentie, Volkan Cakir, Rolf Klemm, Yunhee Kang

**Affiliations:** 1 Helen Keller Intl, Derrière Usine Bastos, 1771 Nv Rte Bastos, Yaoundé, Cameroon; 2 Faculty of Sciences, University of Bamenda, Bamenda, Cameroon; 3 Helen Keller Intl, New York, USA; 4 Faculty of Sciences, University of Yaoundé I, Yaoundé, Cameroon; 5 Department of Public Health, University of Yaoundé I, Yaoundé, Cameroon; 6 Faculty of Life and Earth Sciences, University of Yaoundé I, Yaoundé, Cameroon; 7 Helen Keller Intl, Dakar, Senegal; 8 Helen Keller Intl, Washington, DC USA; 9 International Health Department, Johns Hopkins Bloomberg School of Public Health, Baltimore, USA

**Keywords:** Moderate acute malnutrition (MAM), Food voucher program (FVP), Cameroon, Program evaluation, Recovery

## Abstract

**Background:**

Research on moderate acute malnutrition (MAM) is limited, despite its high prevalence. This study examined outcomes of bi-weekly locally available foods provided via a food voucher program (FVP) on nutritional recovery [mid-upper arm circumference (MUAC) ≥ 125 mm] from MAM (defined as MUAC between 115 and 124 mm) and identified the factors associated with recovery rate in Kaélé health district, Far North Region of Cameroon.

**Methods:**

This was a prospective study with 474 MAM children aged 6–59 months. Food voucher distribution and MUAC screening were conducted at 6 bi-weekly visits or until the child was recovered. Time to recovery was evaluated with multivariate Cox proportional regression hazard models with associations quantified using adjusted hazard ratio (aHR). The trend for MUAC, including its determinants, was examined with multivariate linear mixed effect models.

**Results:**

The recovery rate was 78.3% by 6 weeks after the first food basket; 3.4% remained MAM, and 5.9% were transferred for treatment for severe acute malnutrition (SAM defined as MUAC < 115 mm). Boys were 34% more likely to recover from MAM than girls [aHR = 1.34, 95%CI (1.09, 1.67)]. Children aged 24–53 months were 30% more likely to recover than those aged 6–11 months [aHR = 1.30, 95%CI (0.99, 1.70)]. A one unit increase in weight-for-height Z-score (WHZ) was associated with 1.89-fold greater likelihood of recovery [aHR = 1.89, 95%CI (1.66, 2.14)]. Male children had on average 1.82 mm greater increase in MUAC than female children (*p* < 0.001). One unit increase in WHZ was associated with 3.42 mm increase in MUAC (*p* = 0.025). Children aged 12–23 and 24–53 months had 1.03 mm and 2.44 mm, respectively, greater increase in MUAC over the program than children aged 6–11 months (all *p* < 0.01).

**Conclusion:**

The recovery rate of MAM children treated with the FVP met the Sphere standards for targeted supplementary feeding programs (> 75%). Child’s WHZ, gender and age were significant factors associated with MUAC increase and recovery from MAM in the FVP. These findings indicate the FVP approach shows promise as an effective alternative treatment for MAM with consideration of associated factors and merits further evaluation.

## Background

Malnutrition, in particular low weight-for-height/length, or acute malnutrition, is an important underlying cause of mortality in children [1], and has both acute effects on children’s health and potential long-term effects on their cognitive development and economic growth [2, 3].

Considerable research has focused on the management of severe acute malnutrition [SAM; weight-for-height/length z-scores < − 3 standard deviations (SD) of the 2006 World Health Organization (WHO) growth standards or middle-upper arm circumference (MUAC) < 115 mm] [4–6], while less has addressed treatment for moderate acute malnutrition (MAM; weight-for-height z-scores ≥ − 3 SD and < − 2 SD WHO growth standards or MUAC < 123 and > 115 mm), which is far more prevalent and often a strong predictor of subsequent deterioration to SAM [7].

The prevailing global guidance on the management of MAM dates back to 2012, and prioritizes the use of locally available, nutrient-dense foods [8]. That reference suggests that treatment supports an energy intake of 25 kcal/kg/day in addition to the standard nutrient requirements of a non-malnourished child. Specialized nutritious foods have often been used, particularly in emergencies, to treat moderate wasting. Under the U.S. Agency for International Development (USAID)-funded Food Aid Quality Review project, a study conducted in Sierra Leone investigated the effectiveness and cost-effectiveness of four such products and found equivalent recovery rates over a 12-week period and similar cost-effectiveness, with cost per recovered child ranging from US$90 to US$94 [9].

Cameroon has been coping with a complex humanitarian emergency in its Far North region for nearly a decade, with tens of thousands of refugees and internally displaced persons fleeing conflict and natural resource constraints, exacerbating strains on a fragile health system. The prevalence of SAM and MAM in this region have been reported to be 3.4% and 7.0%, respectively, among the highest in the country although the entire eastern corridor is at alert or precarious levels for multiple nutrition indicators [7]. The prevalence of anemia (hemoglobin concentration < 11.0 g/dl) and chronic malnutrition (height for age < − 2.0 WHO Growth Standards) are also among the country’s highest and very serious at 64% and 37%, respectively, while only about 13% of children 6–23 months of age receive a minimum acceptable diet as defined by WHO (minimum dietary diversity and minimum meal frequency) {Institut National de la Statistique (INS) et ICF, 2020, Enquête Démographique et de Santé du Cameroun 2018}. The few studies that have focused on addressing MAM in this context used ready-to-use supplementary foods (RUSF) or imported food rations under blanket feeding programs.

The government has been testing alternative approaches to increase the coverage and effectiveness of treatment for MAM. In 2016, in coordination with the World Food Programme (WFP), it shifted from targeted supplementary feeding programs (TSFP) to a preventive approach including a ration of Super Cereal Plus for all children aged 6–24 months integrated with complementary services such as social and behavior change communications and reinforced health, water and sanitation services. The TSFP also entailed treating moderate cases among children 6–59 months with Super Cereal Plus. Research found that the new approach reached twice the number of children with greatly reduced cost [10]. Nevertheless, supplies of these imported products are frequently insufficient to meet the needs.

A recent meta-analysis reviewed evidence from three studies that compared recovery rates and weight gain among children with MAM treated with standard RUSF to those treated with local or homemade foods. Evidence suggested that local foods were as beneficial as formulated foods, although the sample size was low and the evidence low quality [11]. A non-peer reviewed study by Action Against Hunger found that food voucher programs implemented in five different contexts to prevent malnutrition improved household dietary diversity in all five programs, reduced acute malnutrition in two, and benefited the participating food vendors [12]. Under the Research on Food Assistance for Nutrition Impact (REFANI) program, a cluster randomized controlled trial in Pakistan found that preventive fresh food vouchers improved weight for height z-scores and reduced the odds of stunting in children aged 6–48 months of age in households receiving them compared to control households. Thus, the limited available evidence suggests the value of further exploring whether supporting household access to local foods can be an effective treatment for moderate wasting. Local procurement has advantages over imported commercially produced RUTF, which have higher financial, transportation, and environmental costs, and have been criticized as medicalizing and commercializing the management of acute malnutrition [13].

In 2017, Helen Keller Intl received a grant from the former USAID Office of Foreign Disaster Assistance to support the treatment of severe and moderate acute malnutrition in seven health districts of Cameroon’s Far North region. When the standard care for MAM, supplementary food supplies from the World Food Programme, could not be secured, the organization developed an alternative approach using vouchers to allow families of children diagnosed with MAM to purchase from local vendors a predefined basket of foods to support rehabilitation. After a promising pilot, the organization received a second grant to implement the program with an operations research component. The aim of this research was to evaluate the fidelity of implementation and the treatment outcomes of a subsample of enrolled children compared to the standards for the management of moderate acute malnutrition defined by the Sphere Project [14].

This paper presents findings on overall recovery rates and the factors associated with recovery and increase in MUAC in children treated under the voucher program.

## Methods

### Study setting

The Far North Region is one of the 10 regions of Cameroon, and is divided into six administrative departments. The project was implemented across four contiguous communes out of the seven communes within the department of Mayo-Kani, where the government identified gaps in coverage with prevention and treatment services. The study was conducted in the commune of Kaélé, which has 14 health areas, a total population of 25,559, and is served by about 70 community health workers (CHW). Each health area encompasses several villages or towns and health facilities (hospitals, clinics, health posts). Agriculture is the economic mainstay, with most people cultivating cereals, mainly sorghum.

### Study design

The study used a pre-post quasi-experimental design to implement a longitudinal cohort study, following and assessing a randomly selected subset of children enrolled in the FVP every 2 weeks from their enrollment in the program until recovery and exit. Children were enrolled in the study with a confirmed MUAC measure ≥ 115 and ≤ 125 mm, and were considered recovered with a MUAC measure > 125 mm. Since previously implemented standard treatment of ready-to-use supplementary foods in the region was no more available, it was not possible to include a comparison group. The study took place between January and August 2020.

### Study participants

Eligible children were identified through a community census and mass screening, during which MUAC of all the children aged 6–59 months in each village or town of the included health districts was measured by the responsible CHW. Children identified as moderately or severely wasted were referred to the nearest health facility, where a public health nurse did further assessments to confirm the diagnosis. Children confirmed as being severely wasted were treated at health facilities following government protocol. Children confirmed with moderate wasting were admitted by health workers into the food voucher program. Children from a randomly selected household were enrolled in the research study following informed consent by the primary caretaker. In households with more than one eligible child, the youngest child was selected for the study. Children were excluded from the study if severely wasted, severely ill, or if the caretakers refuse to give their approval although these children still received the food voucher packages from the FVP program.

### Intervention

**Fig. 1:**
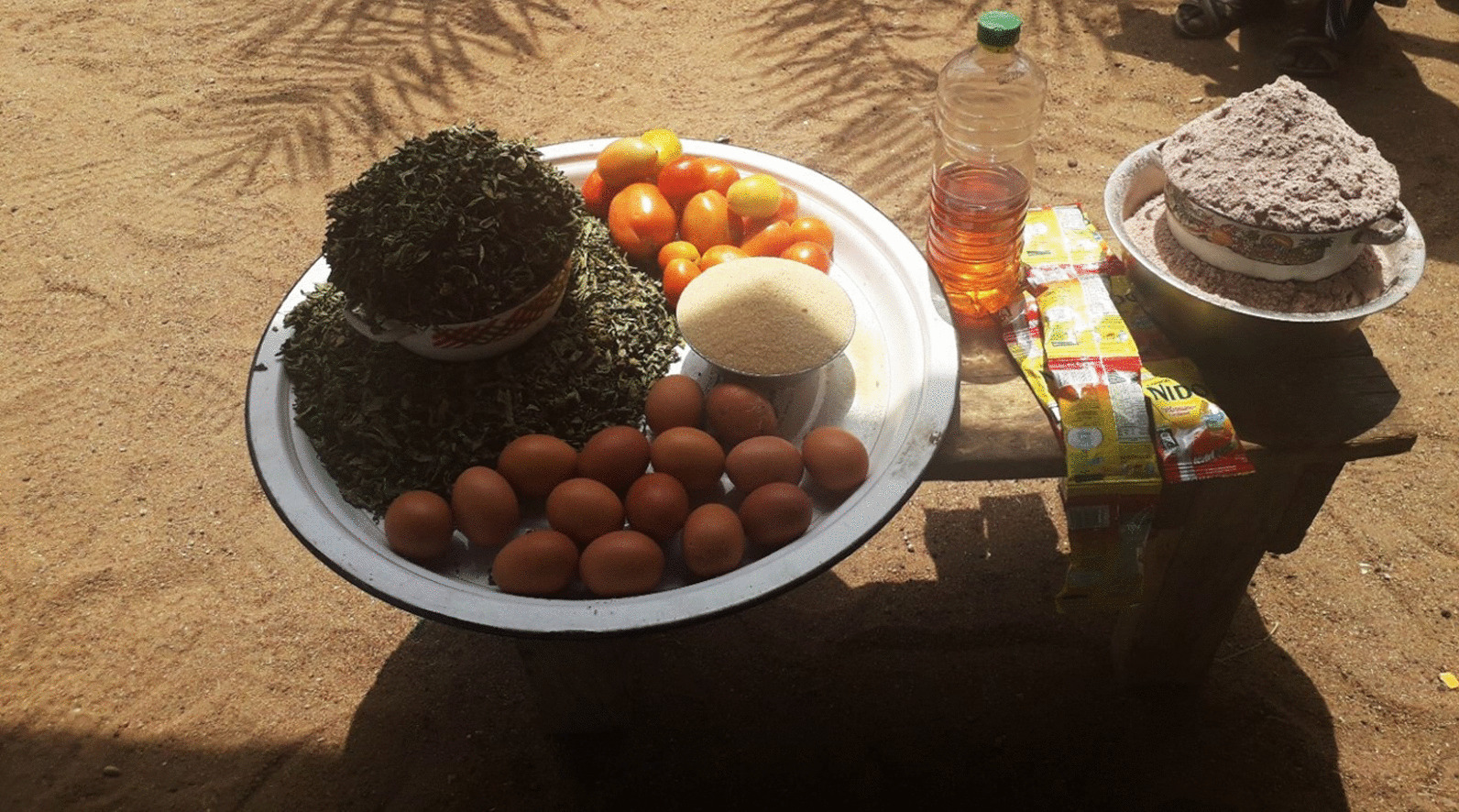
A sample of food basket to be redeemed by an eligible caretaker in Mourbaré district

Once their child was enrolled in the food voucher program by a health facility nurse, caretakers received a paper voucher with a value of 8000 FCFA (~ US$15) they could redeem for a standardized basket of food items from a designated local vendor (Fig. 1). The content of the basket was defined using NutVal software to provide sufficient calories and micronutrients to supplement the usual diet of children and support recovery from moderate wasting, and to provide a small surplus in anticipation of some sharing of the foods within the household (Table 1). The nurse provided instructions to caretakers on the shop or vendor where the voucher was to be redeemed, the composition of the food basket to be received, and to add the ingredients to supplement, not replace, the child’s usual meals. Caretakers also participated in discussion sessions on essential nutrition and hygiene actions, as well as cooking demonstrations of potential recipes to prepare with the voucher foods. These messages were reinforced during bi-weekly home visits by the local CHW to monitor appropriate use of vouchers. Caretakers were instructed to return to the health center with the child every 2 weeks for a health examination and to receive the next voucher.

**Table 1:** Food voucher composition with reference quantity

Item	Reference quantity
Eggs	750 g
Fruits	750 g
Vegetables	750 g
Milk	4500 ml
Sugar	450 g
Oil	450 g
Red millet flour	1500 g

During the program design phase, information gathered through market assessments in the target areas verified that food basket ingredients were readily available in local markets and included a list of local vendors willing and eligible to participate in the program. Eligibility criteria for vendors included their willingness to sign a contract of participation, stock the food items to be redeemed by the voucher and being current with payment of government taxes (including having a tax ID number). The vendors received reimbursement for the vouchers they redeemed every 2 weeks. Monitoring data of voucher redemption and utilization for feeding of the enrolled child indicated that 95% of all vouchers were indeed claimed with vendors.

### Field procedures

Enumerators received 3 days of training on project objectives, questionnaire administration, and anthropometric measurement. Two structured electronic questionnaires (baseline and bi-weekly follow-up) were pretested in French and local dialects with 33 caregivers in neighboring health districts. Each caretaker was explained the nature and objectives of the study and possible risks associated with their participation and provided informed consent before enrollment. Consent was obtained at each follow-up visit as well. Caretakers were allowed to have their questions answered or to refuse any part of the study procedure, or question.

A research team visited the homes of each enrolled child just before their receipt of the first voucher, and every 2 weeks for the duration of the child’s treatment. The baseline survey covered household demographic and socioeconomic characteristics gender-disaggregated asset ownership, agricultural production, food security, health knowledge, child’s recent illness and treatment, and infant and young child feeding knowledge and practices. A 24-h dietary recall was administered by the research team to the caretaker of each enrolled child before entry into the voucher program and at each bi-weekly follow-up visit. Data were collected using the ODK application (www.getodk.org) installed on smartphones and stored on the ONA server (www.ona.io). Local translators were employed to translate the interview from French into the appropriate local language.

The children enrolled in the program were measured every 2 weeks until their exit from the program. In addition to MUAC, three replicates of weight and height/length were recorded at each visit and the mean value calculated. Weight was measured using an electronic scale in kilogram to the nearest decimal fraction with the child standing with both feet in the center of the scale. Children unable or unwilling to stand were weighed in the caretaker’s arms, and the caretaker’s weight was subtracted. All values were reported to the nearest 0.1 kg. Height measurements were reported in centimeters to the nearest decimal fraction using a stadiometer. Children ≥ 2 years and/or ≥ 85 cm were measured for standing height; children < 2 years and/or < 85 cm were measured in the prone position. Date of birth of children was recorded from health card, immunization card or birth certificate to the nearest 0.1 month.

### Outcomes

Children were defined as having recovered when their MUAC was measured as > 125 mm any time of six bi-weekly visits. Those who completed 3 months of treatment without recovery, and those deteriorating during the study period into severe wasting with or without bilateral pitting edema were referred for standard treatment according to the national protocol; specifically, referral to out-patient clinics for further assessment and treatment for SAM. Those who did not return to the health facility for two consecutive treatments were considered by the study as defaulted, although CHW continued to try to reach them to ensure they received appropriate care.

### Sample size and sampling procedure

Based on the total population of 25,199 and 8.3% prevalence of moderate wasting (SMART, 2018) in Kaélé, Far North Region [15], a minimum sample size of *n* = 456 children ensured that a one-sided test with a significance level of 0.05 had 0.90 power to detect a mean difference of 3 mm increase in MUAC by 6 weeks of FVP, assuming a common standard deviation of 15 mm, a Pearson correlation coefficient of 0.01, 2,540 as finite population correction, and a non-response rate of 25% [16]. For the selection of children, a sampling frame was used consisting of the list of confirmed MAM children from the 14 health areas of Kaélé stratified by age and gender, with selection probability proportional to the size of each health area. Although children were also examined biweekly at health centers to review their nutritional status and provide the next voucher per the evaluation, those data were not included in the analyses presented here.

### Statistical methods

Proportions, means (standard deviations), and medians (interquartile ranges) were calculated for key baseline variables for households, mothers, and children. Time to recovery was evaluated with a univariate Cox proportional regression hazard model with associations quantified using hazard ratio (HR) with 95% confidence interval (CI). Covariates significant at ≤ 0.20 in a univariate regression were included and tested in a multivariate Cox proportional hazard model. The trend for MUAC, including its determinants, was studied at univariate level with simple linear fixed and random (mixed) effects models and at multivariate level with multiple linear fixed and random (mixed) effects models, the random variable being the visit. The variables assessed included child anthropometry (MUAC in mm, height in cm, and weight in kg), and demographic and socio-economic characteristics of the caretaker and households. Two-sided *p* values < 0.05 were considered statistically significant in the multivariate models. Data analysis was performed using Stata 16 (StataCorp, 2019) and IBM-SPSS 26 (IBM Corp, 2019).

## Results

Overall in the study area, a total of 28,292 children aged 6–53 months were screened by CHW, and a total of 2126 of these children were confirmed as MAM cases at the health center. A total of 333 agreed to be enrolled in the study. In our study sample, the majority (93.7%) of household heads were married and 43% had more than 6 children (Table 2). About 15% of caretakers had no formal education, with a median age of 27 years (IQR: 23–32). Almost 60% (59.1%) of children were female and were the third or higher child born in the household (59.3%).

**Table 2:** Demographic, anthropometric and socioeconomic characteristics of households, caretakers, and children at enrollment (*n* = 474)

Characteristics	*n*	%
Household characteristics		
Marital status household head		
Monogamy	333	70.3
Polygamy	111	23.4
Single/separated/divorced/widower	30	6.3
Number of children		
≤ 6	270	57.0
> 6	204	43.0
Characteristic of caretaker		
Highest education		
Primary	212	44.7
Secondary or higher	190	40.1
No formal/others	72	15.2
Occupation		
Agriculture	159	33.5
Own business/waged	231	48.7
Housewife	84	17.2
Age, median (IQR)	27	(23, 32)
Child characteristics		
Sex		
Boy	194	40.9
Girl	280	59.1
Age (months)		
6–11	152	32.1
12–23	205	43.2
24–53	117	24.7
Position at household		
1	93	19.6
2	100	21.1
≥ 3	181	59.3

	Mean	SD
WHZ	− 1.90	0.88
MUAC, mm	120	2.64

**Fig. 2:**
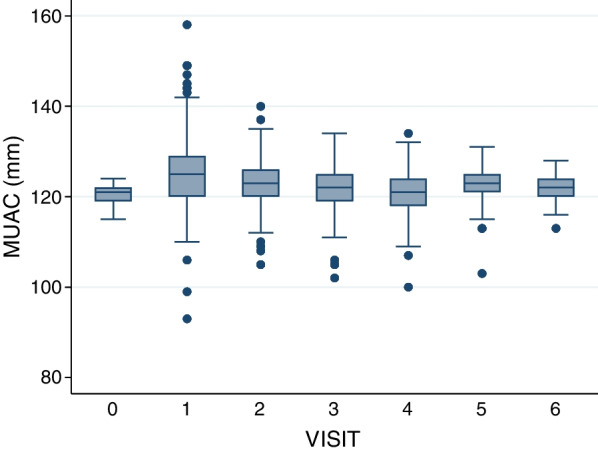
Evolution of children MUAC over visits (every 2-week follow-up)

There was a significant increase in MUAC over time (*p* < 0.001; tested by one-way ANOVA), most pronounced at visits 1 and 2 (2- and 4-week follow-ups, respectively) (Fig. 2).

Overall, the recovery rate of enrolled children was 78.3% (Table 3). A total of 10.5% of children (*n* = 50) deteriorated to SAM and were transferred per government protocol for standard treatment, 3.4% remained moderately wasted by their last follow-up and were also transferred for further treatment, and 7.2% were lost to follow-up. The death rate was 0.2% throughout the program. Most children (71.3%) recovered by visit 3. Indeed, most children recovered early, with 56.1% recovered by visit 1, an additional 36.4% by visit 2, and a further 25.8% by visit 3.

**Table 3:** Treatment outcomes of enrolled children over visits

Visit	Nutritional outcomes					Total
	MAM	Recovered	SAM	Death	Defaulted	
	*N* (%)	*N* (%)	*N* (%)	*N* (%)	*N* (%)	*N* (%)
0	474 (100.0)	0 (0.0)	0 (0.0)	0 (0.0)	0 (0.0)	474 (100.0)
1	153 (37.5)	229 (56.1)	17 (4.2)	1 (0.2)	8 (2.0)	408 (100.0)
2	113 (52.8)	78 (36.4)	10 (4.7)	0 (0.0)	13 (6.1)	214 (100.0)
3	71 (59.2)	31 (25.8)	10 (8.3)	0 (0.0)	8 (6.7)	120 (100.0)
4	53 (66.2)	17 (21.3)	9 (11.3)	0 (0.0)	1 (1.2)	80 (100.0)
5	27 (58.9)	12 (26.1)	3 (6.5)	0 (0.0)	4 (8.7)	46 (100.0)
6	15 (75.0)	4 (20.0)	1 (5.0)	0 (0.0)	0 (0.0)	20 (100.0)
Overall^a^	15 (3.2)	371 (78.3)	50 (10.5)	1 (0.2)	34 (7.2)	474 (100.0)

^a^By including three missing cases (0.6%), the total percentages add up to 100%

In the bivariate Cox regression model, only higher WHZ at each visit of assessment was found to be a highly significantly associated with recovery (HR = 2.33, 95%CI (2.06, 2.63), *p* = 0.000) (Table 4). WHZ, child age, and child sex, cohort, and maternal education were included in the multivariate Cox regression model. The probability of recovery from MAM was higher among children 24–53 months of age compared to those aged 6–11 months [aHR = 1.31 95%CI (1.00, 1.73)]. Boys showed a 33% higher rate of recovery compared to girls [aHR = 1.33, 95%CI (1.08, 1.65)]. A one unit increase in WHZ was significantly associated with 1.89-fold higher recovery rate [aHR = 1.89, 95%CI (1.66, 2.14)].

**Table 4:** Cox proportional hazards model of factors associated with recovery from moderate acute malnutrition

	Univariate		Multivariate	
	HR (95%CI)	*p* value	HR (95%CI)	*p* value
Child sex (ref: Female)	1.00		1.00	
Male	1.09 (0.89, 1.34)	0.41	1.33 (1.08, 1.65)	0.008
Child age (ref: 6–11 mo)	1.00		1.00	
12–23 months	1.13 (0.88, 1.45)	0.35	1.10 (0.86, 1.42)	0.47
24–53 months	1.43 (1.09, 1.87)	0.01	1.31 (1.00, 1.73)	0.05
WHZ	1.82 (1.61, 2.06)	< 0.001	1.89 (1.66, 2.14)	< 0.001
Season (Ref: dry season)	1.00		1.00	
Rainy season	0.97 (0.79, 1.19)	0.76	1.16 (0.93, 1.43)	0.18
Mother’s education (ref: no formal education)	1.00		1.00	
Primary	1.28 (0.94, 1.76)	0.12	1.27 (0.92, 1.74)	0.14
Secondary or higher	1.14 (0.83, 1.58)	0.41	1.25 (0.90, 1.73)	0.18
Mother’s occupation (ref: housewife)	1.00			
Agriculture	1.11 (0.82, 1.50)	0.52		
Own business/waged job	1.19 (0.89, 1.59)	0.23		
Family size (ref: ≤ 6)	1.00			
> 6	0.94 (0.77, 1.16)	0.59		

At the bivariate level using simple linear fixed and random (mixed) effects models, child sex, age, and WHZ had *p* ≤ 0.2, and thus were included in a multiple linear fixed and random (mixed) effects models (Table 5). One unit increase in WHZ was associated with 3.42 mm higher MUAC (*p* < 0.001). Children aged 12–23 and 24–53 months had on average 1.03 mm and 2.44 mm greater increase in MUAC than those aged 6–11 months (*p* < 0.01).

**Table 5:** Linear Fixed and Random (Mixed) effects models of factors associated with MUAC

	Univariate		Multivariate	
	*β* (95%CI)	*p* value	*β* (95%CI)	*p* value
Child sex (ref: Female)	–		–	
Male	0.48 (− 0.22, 1.17)	0.18	1.82 (1.23, 2.40)	< 0.001
Child age (ref: 6–11 months)	–		–	
12–23 months	1.03 (0.27, 1.80)	0.008	1.03 (0.38, 1.68)	0.002
24–53 months	2.598 (1.68, 3.48)	< 0.001	2.44 (1.68, 3.20)	< 0.001
WHZ	3.23 (2.91, 3.55)	< 0.001	3.42 (3.10, 3.74)	< 0.001
Season (ref: Dry season)	–		–	
Rainy season	− 0.07 (− 1.23, 1.09)	0.91	0.80 (− 0.20, 1.78)	0.116
Mother’s education (ref: no formal education)	–			
Primary	0.45 (− 0.66, 1.56)	0.43		
Secondary or higher	0.02 (− 1.11, 1.16)	0.97		
Mother’s occupation (ref: housewife)	–			
Agriculture	− 0.17 (− 1.21, 0.88)	0.76		
Own business/waged job	− 0.06 (− 1.02, 0.92)	0.91		
Family size (ref: ≤ 6)	–			
> 6	0.43 (− 0.53, 1.40)	0.38		

## Discussion

The objective of this study was to assess recovery among children enrolled in a novel food voucher program and evaluate additional factors associated with recovery rate and the time to recovery. Time to recovery and recovery rates were significantly affected by WHZ, child age and gender. Weight-for-height z-score was found to be associated with MUAC increase and greater rate of recovery, which is unsurprising as the two indicators are correlated. It is important to point out research showing that while correlated, these indicators more often identify different rather than the same children as suffering from wasting [17]. An analysis of surveys from 48 countries covering over 1.3 million children found that on average only 28.2% of wasted children (MUAC < 125 mm or WHZ < − 2 SD) received the same diagnosis by both measures, with a range between 15 and 38.5% [18]. The authors also found great variation among countries in the relative sensitivity of each measure and recommended both indicators be used together to be sure all children receive appropriate care. Nevertheless, it is not surprising that a higher WHZ was associated with shorter time to recovery.

The recovery, default, and death rates observed in this study were within the acceptable range of Global Sphere standards for moderate acute malnutrition, namely: mortality < 3%, default < 15% and recovered > 75% [19]. According to the Sphere Handbook, default and coverage rates can be used as proxy indicators for the acceptability of a program (p. 176), suggesting good acceptability of the food voucher program. Interview data collected under the study indicated that default in our context was mainly due to marital conflict and family relocation. The recovery rate under this food voucher treatment was also comparable to that reported for children treated with Corn Soy Blend Plus [73% (95% CI 59%, 87%)] and RUSF [85% (95% CI 73%, 97%)] in Cameroon by Medoua et al. [20] under a randomized controlled trial.

A comprehensive review of the impact of complementary feeding interventions on key child health and nutritional outcomes conducted a number of years ago revealed the complexity of the challenges in improving the adequacy of young child diets, especially in low-income settings, but suggested that the provision of foods together with nutrition education has shown a greater impact on child weight gain and growth than either intervention alone, or than fortifying or increasing the energy density of children’s usual diets [21]. The review did not examine programs addressing child wasting specifically. Our program included a behavior change communications component delivered through group discussions and individual household visits, which data to be reported separately suggested reinforced the appropriate use of the nutritious foods for child feeding. A more recent review compared outcomes from programs providing counseling on child feeding alone to those providing formulated food supplements and found greater recovery among children treated with food products [22]. Those authors also highlighted the dearth of high quality and comparable studies and the difficulty drawing conclusions from the existing body of literature. They were able to identify only one study that provided a ration of local foods to children with moderate wasting consisting of rice, wheat, lentils and oil. The calorie-rich supplement had mixed results, improving weight gain during the first 3 months of the program but not over the second 3 months. The authors of that study suggested the weakness of the ration was its lack of micronutrient density [23]. Our food basket was designed to supplement both caloric and micronutrient intake of enrolled children.

Also of relevance are three studies under the REFANI consortium designed to evaluate a range of strategies for the prevention of wasting in communities facing humanitarian emergencies [24]. That research examined different levels of cash transfer, and, in one setting, a fresh food voucher, provided during seasons of high food insecurity. In a longitudinal, cluster randomized controlled trial conducted in Pakistan [25], the voucher, like ours, had a value of approximately US$14 and was redeemed for a specified list of fresh foods in preselected shops. Unlike our study, the vouchers were preventative rather than a treatment for wasting. The study found that, compared to children in the control arm, children receiving the food voucher did not have a reduced risk of moderate wasting (weight-for-height z-score < 2 SD WHO norms) at the end of the 6 months of the intervention or at 1 year, but they did have significantly higher mean WHZ at 6 months (although the effect disappeared at 1 year). Children in the arm receiving a double cash transfer did show both reduced risk of wasting and improved mean WHZ at 6 months compared to the controls, but those gains were also no longer detectable at 1 year. Surprisingly, all three interventions (including a single cash transfer) significantly improved child linear growth, a secondary outcome, at both time points and to a similar degree. The food voucher group also unexpectedly experienced reduced mean hemoglobin concentration at the 6-month assessment, and lower improvement in dietary diversity and animal-source food consumption compared to the control group than the other intervention arms. The authors reported qualitative data suggesting the food vouchers (and how they were interpreted by food vendors) allowed households less flexibility in food choice than did the interventions providing cash. While our study did not include a counterfactual, our data did show a significant increase in dietary diversity among enrolled children compared to baseline (manuscript in preparation).

In our study, boys were more likely to recover from moderate acute malnutrition than girls. Similar results were obtained in a study of treatment outcomes of children with severe wasting in Ethiopia [26] and in a trial in Burkina Faso of a simplified protocol for managing severe and moderate wasting [27]. By contrast, a longitudinal follow-up study of children in Malawi treated for moderate wasting with corn-soy blend (CSB)++ or RUSF found that female children were less likely to relapse at 3 or 12 months [28]. That study also found an alarming level of relapse to moderate (17%) and severe (10%) wasting, as well as a 4% mortality rate, underscoring how vulnerable wasted children remain, even after successful recovery.

### Limitations

Given the lack of standard treatment for MAM in the study setting, we were not able to include a comparison arm against which to assess the performance of the FVP. We also have no follow-up data to evaluate whether children who recovered were able to maintain their nutritional status after the discontinuation of vouchers. Our findings could be generalized to other settings, where local food supplies are easy to access, less pressures on households to share foods with other family members.

## Conclusion

Findings from our study indicate that a voucher program to enable families to purchase locally available foods shows promise in its acceptability and support for recovery. Further research is needed based on scaled-up programs and for a rigorous cost analysis to determine the cost-effectiveness of this approach.

## Data Availability

The datasets generated and/or analyzed during the current study are not publicly available but are available from the corresponding author on reasonable request.

## Abbreviations

FVP: Food voucher program
MAM: Moderate acute malnutrition MAM
MUAC: Mid-upper arm circumference
RUSF: Ready-to-use supplementary foods
WHZ: Weight-for-height Z-score
